# HIV treatment is associated with a twofold higher probability of raised triglycerides: pooled analyses in 21 023 individuals in sub-Saharan Africa

**DOI:** 10.1017/gheg.2018.7

**Published:** 2018-05-08

**Authors:** K. Ekoru, E. H. Young, D. G. Dillon, D. Gurdasani, N. Stehouwer, D. Faurholt-Jepsen, N. S. Levitt, N. J. Crowther, M. Nyirenda, M. A. Njelekela, K. Ramaiya, O. Nyan, O. O. Adewole, K. Anastos, C. Compostella, J. A. Dave, C. M. Fourie, H. Friis, I. M. Kruger, C. T. Longenecker, D. P. Maher, E. Mutimura, C. E. Ndhlovu, G. Praygod, E. W. Pefura Yone, M. Pujades-Rodriguez, N. Range, M. U. Sani, M. Sanusi, A. E. Schutte, K. Sliwa, P. C. Tien, E. H. Vorster, C. Walsh, D. Gareta, F. Mashili, E. Sobngwi, C. Adebamowo, A. Kamali, J. Seeley, L. Smeeth, D. Pillay, A. A. Motala, P. Kaleebu, M. S. Sandhu, the African Partnership for Chronic Disease Research (APCDR)

**Affiliations:** 1 Department of Medicine, University of Cambridge, Cambridge, UK; 2 Global Health and Populations Group, Wellcome Trust Sanger Institute, Hinxton, Cambridge, UK; 3 Weill Cornell Medical College, New York City, New York, USA; 4 University Hospitals Case Medical Center, Cleveland, Ohio, USA; 5 Department of Infectious Diseases, University of Copenhagen (Rigshospitalet), Copenhagen, Denmark; 6 Division of Diabetic Medicine and Endocrinology, Department of Medicine, University of Cape Town, Cape Town, South Africa; 7 Department of Chemical Pathology, National Health Laboratory Service, University of the Witwatersrand Medical School, Johannesburg, South Africa; 8 Malawi Epidemiology and Intervention Research Unit, Malawi, Lilongwe; 9 Department of Physiology, Muhimbili University of Health and Allied Sciences, Dar es Salaam, Tanzania; 10 Shree Hindu Mandal Hospital, Dar es Salaam, Tanzania; 11 Royal Victoria Teaching Hospital, School of Medicine, University of The Gambia, Banjul, The Gambia; 12 Department of Medicine, Obafemi Awolowo University, Ile Ife, Nigeria; 13 Albert Einstein College of Medicine, Bronx NY, USA; 14 Department of Medicine, University of Padua, Padua, Italy; 15 Division of Diabetic Medicine and Endocrinology, Department of Medicine, University of Cape Town, Cape Town, South Africa; 16 HART (Hypertension in Africa Research Team), North-West University, Potchefstroom, South Africa; 17 Department of Nutrition, Exercise and Sports, Faculty of Science, University of Copenhagen, Denmark; 18 Africa Unit for Transdisciplinary Health Research (AUTHeR), North-West University, Potchefstroom, South Africa; 19 Special Programme for Research & Training in Tropical Diseases (TDR), World Health Organization, Geneva, Switzerland; 20 Clinical Epidemiology Resource Training Centre, University of Zimbabwe College of Health Sciences, Harare, Zimbabwe; 21 National Institute for Medical Research, Tanzania, Dar es Salaam; 22 Chest Unit of Yaounde Jamot Hospital, Cameroon, Yaoundé; 23 Epicentre, Médecins Sans Frontières, Paris, France; 24 Department of Epidemiology and Public Health, University College of London, Clinical Epidemiology Group, London, UK; 25 Cardiology Unit, Department of Medicine, Aminu Kano Teaching Hospital, Kano, Nigeria; 26 MRC Unit for Hypertension and Cardiovascular Disease, North-West University, Potchefstroom, South Africa; 27 Soweto Cardiovascular Research Unit, Chris Hani Baragwanath Hospital, University of the Witwatersrand, Johannesburg, South Africa; 28 Department of Medicine, University of California, San Francisco, USA; 29 Faculty of Health Sciences, North-West University, Potchefstroom, South Africa; 30 Department of Nutrition and Dietetics, University of the Free State, Bloemfontein, South Africa; 31 Africa Health Research Institute, University of KwaZulu-Natal, Durban, South Africa; 32 Faculty of Medicine and Biomedical Sciences, University of Yaoundé 1, Cameroon, Yaoundé; 33 Institute of Human Virology, Abuja, Nigeria; 34 Department of Epidemiology and Public Health, Institute of Human Virology and Greenebaum Cancer Center, University of Maryland School of Medicine, Baltimore, USA; 35 MRC/UVRI Uganda Research Unit on AIDS, Entebbe, Uganda; 36 Faculty of Epidemiology and Population Health, London School of Hygiene and Tropical Medicine, London, UK; 37 Department of Diabetes and Endocrinology, Nelson R. Mandela School of Medicine, University of KwaZulu-Natal, Durban, South Africa

**Keywords:** Antiretroviral therapy, cardiovascular disease, HIV, lipids, sub-Saharan Africa, triglycerides

## Abstract

**Background:**

Anti-retroviral therapy (ART) regimes for HIV are associated with raised levels of circulating triglycerides (TGs) in western populations. However, there are limited data on the impact of ART on cardiometabolic risk in sub-Saharan African (SSA) populations.

**Methods:**

Pooled analyses of 14 studies comprising 21 023 individuals, on whom relevant cardiometabolic risk factors (including TG), HIV and ART status were assessed between 2003 and 2014, in SSA. The association between ART and raised TG (>2.3 mmol/L) was analysed using regression models.

**Findings:**

Among 10 615 individuals, ART was associated with a two-fold higher probability of raised TG (RR 2.05, 95% CI 1.51–2.77, I2 = 45.2%). The associations between ART and raised blood pressure, glucose, HbA1c, and other lipids were inconsistent across studies.

**Interpretation:**

Evidence from this study confirms the association of ART with raised TG in SSA populations. Given the possible causal effect of raised TG on cardiovascular disease (CVD), the evidence highlights the need for prospective studies to clarify the impact of long term ART on CVD outcomes in SSA.

## Background

Epidemiological studies of environmental and genetic risk factors indicate that elevated triglycerides (TGs), remnant cholesterol or TG-rich lipoproteins may be causal risk factors for cardiovascular disease (CVD) [1–3]. Anti-retroviral therapy (ART) is associated with dyslipidaemia, including increased levels of circulating TGs in populations of European decent [4–6]. As such, long-term ART may be associated with increased risk of CVD. Indeed, observational evidence suggests that certain ART regimens may be associated with increased risk of CVD in European populations [7].

However, in sub-Saharan Africa (SSA), a region with the highest burden of HIV and where access to ART has substantially increased over the last decade, the association between ART and TGs has not been clarified. Importantly, the relationship between ART and risk factors for CVD in populations from SSA may be more complex because of differences in cardiometabolic risk profiles, HIV strains, efficacy of ART and environmental factors [8–19]. It is therefore crucial to assess the association between ART and cardiovascular risk factors in SSA populations – to inform strategies to control the rising burden of CVD in the region.

We previously conducted a systematic review to provide a preliminary assessment of the relationship between HIV and ART with a set of cardiometabolic risk factors [20]. In this paper, we extend this work to synthesize existing evidence using individual participant data (IPD) pooled analyses to more reliably assess the magnitude and direction of association between ART and raised TGs and other risk factors for cardiometabolic disease in SSA.

## Methods

### Data sources and inclusion criteria

We invited 57 investigators to contribute IPD for pooled analysis: 52 investigators who had collaborated in a previous systematic review of the association between HIV, ART and cardiometabolic risk factors [20], and five investigators identified through personal communication with other collaborators. Briefly, we included studies conducted in SSA that had collected data on HIV/ART status and the relevant cardiometabolic risk factors among black Africans aged 13 years or older. Studies were excluded if they lacked a comparison group, or had too few (<10) or no participants with a risk factor based on a defined cut-off, or had a very small sample size (<10 participants).

### Data collation

We requested data on cardiometabolic risk factors [blood pressure (BP), lipids, glucose and glycated haemoglobin (HbA1c)], HIV infection and ART status. We also collected additional variables for adjustment (Table 1). Data were checked for plausibility and consistency and, when necessary, collaborators were contacted for clarification before analysis. We converted all variables measured on other scales to the SI scale (mmol/L, %, mm Hg or Kg/m^2^).

**Table 1.** Data requested for estimating the magnitude and direction of association between anti-retroviral therapy (ART) and selected cardiometabolic risk factors in sub-Saharan Africa

Cardiometabolic risk factor	HIV and ART information	Additional information
TG level	HIV status	Sex
Total cholesterol level	Date of first positive HIV test	Age
HDL level	Date of last negative HIV test	Country of origin
LDL level	WHO HIV stage	Ethno-linguistic group
BMI	Serial CD4 counts with dates	Education level
SBP	Serial viral load measurements with dates	Smoking history
DBP	ART status	Alcohol consumption
Blood glucose level (FBG/RBG)	Date of ART initiation	Hepatitis B status
HbA1c level	Type of ART with dates of use	Hepatitis C status
Lipid-lowering medication and duration, glucose-lowering medication and duration, blood pressure-lowering medication and duration		Date of blood draw for lipid measurement

TG, triglycerides; HDL, high-density lipoprotein cholesterol; LDL, low-density lipoprotein cholesterol; BMI, body mass index; SBP, systolic blood pressure; DBP, diastolic blood pressure; FBG, fasting blood glucose; RBG, random blood glucose; HbA1c, glycated haemoglobin; ART, antiretroviral therapy; WHO, World Health Organization.

### Definition of outcomes and exposure

The outcomes for this study were binary cardiometabolic risk factors defined according to predefined clinical cut-offs [21–26]. We analysed the following risk factors: raised TGs (>2.3 mmol/L), raised low-density lipoprotein (LDL ⩾3.3 mmol/L), raised total cholesterol (TC >5.2 mmol/L) and low high-density lipoprotein (HDL <1.3 mmol/L for women and HDL <1.0 mmol/L for men). We also analysed raised BP (>140/90 mm Hg), raised blood glucose (fasting blood glucose ⩾7.0 mmol/L or random blood glucose ⩾11.1 mmol/L) and raised HbA1c (⩾6.5%). The exposures of interest were HIV infection status (positive or negative) and ART use. HIV infection status was defined as presented in each study. An individual was considered to have untreated HIV infection if they were HIV-positive and had never received ART medication. In addition, individuals recorded as receiving ART were considered to be HIV-infected. We defined ART use as a receipt of ART medication at the time of cardiometabolic risk factor measurement.

### Statistical analysis

We conducted a two-step IPD pooled analysis, analysing each dataset separately to obtain study-level estimates, before combining them using random-effects models of meta-analysis. We fitted Poisson regression models with robust sandwich estimators of variance for each outcome to obtain study-specific risk ratios (RRs) and prevalence ratios (PRs) for ART use and untreated HIV infection [27, 28]. RRs and PRs are collectively referred to as RR hereafter. We used multilevel mixed models to adjust for clustering of individuals within households in two cross-sectional studies and to account for correlation in repeated measurements in one longitudinal study included in the analyses.

In our primary analysis, we assessed the association between ART and each cardiometabolic risk factor by comparing individuals receiving ART (ART+) with individuals not receiving ART. Individuals not receiving ART were either untreated HIV-positive individuals (in studies of HIV-positive individuals only) or a combination of untreated HIV-positive individuals and HIV-negative (HIV-) individuals (in studies including both groups). In sensitivity analyses, we also compared associations between individuals receiving ART and HIV-negative individuals, and between individuals receiving ART and untreated HIV-positive individuals. We also compared untreated HIV-positive individuals with HIV-negative individuals to assess the impact of HIV infection on cardiometabolic risk independent of ART use.

All models were adjusted for body mass index (BMI), age and sex. In a subgroup of studies where data were available, we also adjusted for alcohol consumption, current smoking status, education level, fruit and vegetable consumption, physical activity and socio-economic position. Additionally, where data were available for lipid and glucose outcomes, we adjusted for lipid- and glucose-lowering medication, respectively. For BP as an outcome, we adjusted for BP-lowering medication where data were available. Further, for each cardiometabolic risk factor studied as an outcome variable, the other cardiometabolic risk factors were additionally adjusted for.

To pool the adjusted RRs, we estimated a weighted average of study-specific log (adjusted RR) incorporating between-study heterogeneity according to the method of DerSimonian and Laird [29]. The *I*^2^ statistic was used to assess the heterogeneity between study-specific estimates [30].

A predetermined set of study-level characteristics were assessed as potential sources of heterogeneity between studies using meta-regression: study type (population-based, clinic-based), study size, year the study was conducted, location of study (West, East, Central or Southern Africa), sex distribution (proportion of men), mean participant BMI and mean participant age. Lastly, we conducted sensitivity analyses to assess whether a single study could have influenced pooled RR results excessively by excluding each study from the pooled analysis in turn, and comparing results with and without the study in question.

All analyses were performed using STATA 13.1 (Stata, College Station, TX, USA).

### Ethics

This study received ethical approval from the Human Biology Research Ethics Committee at the University of Cambridge, UK (Application No: HBREC.2015.05), and each primary study obtained informed consent from participants.

## Results

**Fig. 1.**
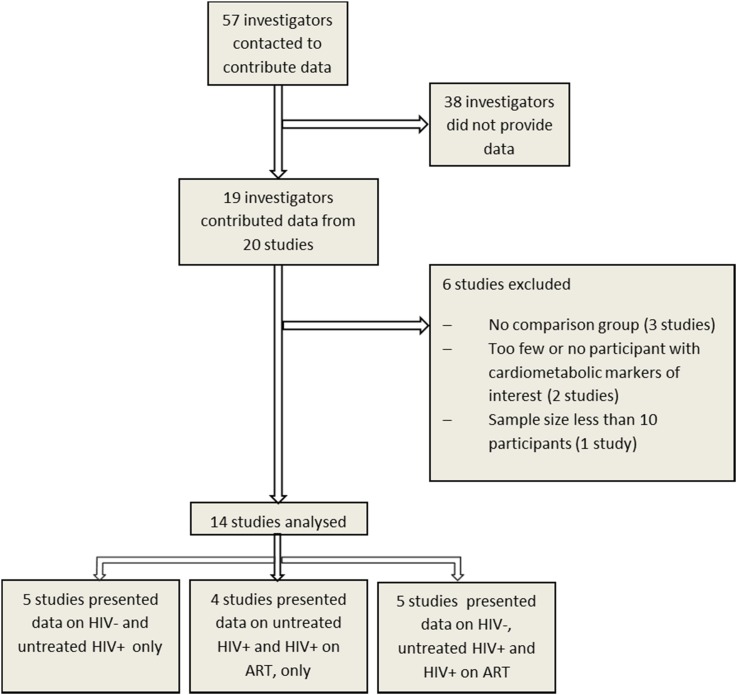
Study selection for individual participant data pooled analysis to assess the association of HIV and anti-retroviral therapy with cardiometabolic risk in sub-Saharan Africa.

We received data for 20 studies and included 14 studies conducted between 2003 and 2014 in the current analysis (Fig. 1). Overall, the pooled data comprised 21 023 participants aged 13–107 years, with generally fewer men than women but with varying proportions for each cardiometabolic risk factor studied (Table 2). The number of individuals and proportion of men and women included in the analyses varied by cardiometabolic risk factors because not all studies had data on all risk factors, and because of missing data within studies. Apart from one all-women study, the proportion of men across all studies ranged between 22% and 49%. In the primary analyses (comparing individuals on ART to individuals not receiving ART), the number of participants included ranged from 6364 with data on glucose to 10 620 with data on TC (Table 2).

**Table 2.** Characteristics of 14 studies included in the pooled analyses to assess the association between HIV/anti-retroviral therapy (ART) and selected cardiometabolic risk factors in sub-Saharan Africa

							Number of participants			
Risk factor	Study/investigator	Study period	Study type	Location	Age range	Percentage of men	HIV-negative	HIV-positive	Untreated HIV-positive	on ART
Raised TG^a^	THUSA^42^	2004	Population based	Southern Africa	15–90	42	1440	204	0	0
	Sani^43^	2005	Clinic based	West Africa	20–50	47	0	0	100	100
	Schutte^44^	2007	Population based	Southern Africa	20–77	49	251	108	0	0
	Mutimura^45^	2007	Clinic based	East Africa	25–70	0	112	361	0	0
	Dave^46^	2008	Clinic based	Southern Africa	19–68	22	0	0	404	551
	Stehouwer^47^	2008	Clinic based	East Africa	18–74	34	0	0	379	191
	Kruger-Fourie^48^	2010	Population based	Southern Africa	35–98	35	850	168	107	61
	Walsh^49^	2011	Population based	Southern Africa	25–65	22	669	254	209	45
	GPC^50^	2011	Population based	East Africa	17–100	43	4954	547	339	208
	Pefura^51^	2011	Clinic based	West Africa	18–67	40	0	0	138	204
	DDS^52^	2014	Population based	Southern Africa	18–91	29	599	507	315	192
**Total**							**8875**	**2149**	**1991**	**1552**
Raised LDL^b^	THUSA	2004	Population based	Southern Africa	15–90	42	1421	201	0	0
	Sani	2005	Clinic based	West Africa	20–50	47	0	0	100	100
	Schutte	2007	Population based	Southern Africa	20–77	49	249	107	0	0
	Mutimura	2007	Clinic based	East Africa	25–70	0	106	316	0	0
	Dave	2008	Clinic based	Southern Africa	19–68	22	0	0	403	550
	Stehouwer	2008	Clinic based	East Africa	18–74	37	0	0	337	192
	Kruger-Fourie	2010	Population based	Southern Africa	35–98	35	850	168	107	61
	Walsh	2011	Population based	Southern Africa	25–65	22	661	254	209	44
	GPC	2011	Population based	East Africa	17–100	43	4954	547	339	208
	Pefura	2011	Clinic based	West Africa	18–67	40	0	0	138	204
	DDS	2014	Population based	Southern Africa	18–91	29	599	507	315	192
**Total**							**8840**	**2100**	**1948**	**1551**
Low HDL^c^	THUSA	2004	Population based	Southern Africa	15–90	42	1464	206		
	Sani	2005	Clinic based	West Africa	20–50	47	0	0	100	100
	Schutte	2007	Population based	Southern Africa	20–77	49	251	108	0	0
	Mutimura	2007	Clinic based	East Africa	25–70	0	184	511	0	0
	Dave	2008	Clinic based	Southern Africa	19–68	22	0	0	404	551
	Stehouwer	2008	Clinic based	East Africa	18–74	36	0	0	338	181
	Kruger-Fourie	2010	Population based	Southern Africa	35–98	35	851	168	107	61
	Walsh	2011	Population based	Southern Africa	25–65	22	669	254	209	45
	GPC	2011	Population based	East Africa	17–100	43	4954	547	339	208
	Pefura	2011	Clinic based	West Africa	18–67	40	0	0	138	204
	DDS	2014	Population based	Southern Africa	18–91	29	599	507	315	192
**Total**							**8972**	**2301**	**1950**	**1542**
Raised TC^d^	THUSA	2004	Population based	Southern Africa	15–90	42	1439	204	0	0
	Sani	2005	Clinic based	West Africa	20–50	47	0	0	100	100
	Schutte	2007	Population based	Southern Africa	20–77	49	251	108	0	0
	Mutimura	2007	Clinic based	East Africa	25–70	0	183	475	0	0
	Dave	2008	Clinic based	Southern Africa	19–68	22	0	0	404	551
	Stehouwer	2008	Clinic based	East Africa	18–74	35	0	0	381	195
	Kruger-Fourie	2010	Population based	Southern Africa	35–98	35	850	167	106	61
	Walsh	2011	Population based	Southern Africa	25–65	22	669	254	209	45
	GPC	2011	Population based	East Africa	17–100	43	4954	547	339	208
	Pefura	2011	Clinic based	West Africa	18–67	40	0	0	138	204
	DDS	2014	Population based	Southern Africa	18–91	29	599	507	315	192
**Total**							**8945**	**2262**	**1992**	**1556**
Raised blood pressure^e^	Africa Centre (2003)^53^	2003	Population based	Southern Africa	17–72	32	1435	649	0	0
	THUSA	2004	Population based	Southern Africa	15–90	42	1505	209	0	0
	Sani	2005	Clinic based	West Africa	20–50	47	0	0	100	100
	Schutte	2007	Population based	Southern Africa	20–77	49	258	112	0	0
	Mutimura	2007	Clinic based	East Africa	25–70	0	187	536	0	0
	Dave	2008	Clinic based	Southern Africa	19–68	22	0	0	391	547
	Stehouwer	2008	Clinic based	East Africa	16–72	35	0	0	409	306
	Kruger-Fourie	2010	Population based	Southern Africa	35–98	35	872	169	108	61
	Africa Centre (2010)^53^	2010	Population based	Southern Africa	14–107	31	5752	1823	0	0
	Walsh	2011	Population based	Southern Africa	25–65	22	667	250	206	44
	GPC	2011	Population based	East Africa	17–100	43	4945	548	337	211
	DDS	2014	Population based	Southern Africa	18–91	29	599	507	315	192
**Total**							** 16 220**	**4803**	**1866**	**1461**
Raised blood glucose^f^	THUSA	2004	Population based	Southern Africa	15–90	42	1421	204	0	0
	Sani	2005	Clinic based	West Africa	20–50	47	0	0	100	100
	Schutte	2007	Population based	Southern Africa	20–77	49	251	108	0	0
	Mutimura	2007	Clinic based	East Africa	25–70	0	188	536	0	0
	Dave	2008	Clinic based	Southern Africa	19–68	22	0	0	404	551
	Stehouwer	2008	Clinic based	East Africa	18–76	32	0	0	93	151
	Faurholt-Jepsen^54^	2009	Clinic based	East Africa	13–89	55	1227	677	597	80
	Kruger-Fourie^g^	2010	Population based	Southern Africa	35–98	35	859	168	107	61
	Walsh^g^	2011	Population based	Southern Africa	25–65	22	677	251	206	45
	DDS	2014	Population based	Southern Africa	18–91	29	599	507	315	192
**Total**							**5222**	**2451**	**1822**	**1180**
Raised HbA1c^h^	Kruger-Fourie	2010	Population based	Southern Africa	35–98	35	863	168	107	61
	Walsh^g^	2011	Population based	Southern Africa	25–65	22	681	253	208	45
	GPC^g^	2011	Population based	East Africa	17–97	43	4939	546	339	207
	DDS	2014	Population based	Southern Africa	18–91	29	599	507	315	192
**Total**							**7082**	**1474**	**969**	**505**

TG, triglycerides; LDL, low-density lipoprotein; HDL, high-density lipoprotein; TC, total cholesterol; HbA1c, glycated haemoglobin.

a Raised TG defined as TG >2.3 mmol/L.

b Raised LDL defined as LDL ⩾3.3 mmol/L.

c Low HDL defined as HDL <1.3 mmol/L (women) and HDL <1.0 mmol/L (men).

d Raised TC defined as TC >5.2 mmol/L.

e Raised BP defined as systolic blood pressure ⩾140 mm Hg or diastolic blood pressure ⩾90 mm Hg.

f Raised glucose defined as glucose ⩾7.0 mmol/L (fasting) or glucose ⩾11.1 mmol/L (non-fasting).

g Estimates for ART not available because of too few cases of raised glucose; GPC, General Population Cohort; THUSA, Transition and Health during Urbanization in South Africa; DDS, Durban Diabetes Study.

h Raised HbA1c defined as HbA1c ⩾6.5%.

From the above, 10 615 [36% men; age range 17–100 years; mean age 41.4 years (SD 14.0)] individuals from eight studies provided data for analyses of the association between ART and raised TG. Of these, 1552 were on ART, 1413 (91%) of whom provided data on ART regimen (Table 3). Among those with data on ART regimen, 80% were on two nucleoside reverse transcriptase inhibitors (NRTIs) [mainly zidovudine (AZT) and lamivudine, 80%] and one non-nucleoside reverse transcriptase inhibitor (NNRTI) [mainly efavirenz (EFV) or nevirapine (NVP), 87%] (two NRTIs + one NNRTI); while 13% received two NRTIs and one protease inhibitor (PI) (two NRTIs + one PI) (Table 3). In all, 87% of the individuals with TG data, who were on ART, and provided ART regimen data, were on a non-PI combination (predominantly, two NRTIs + one NNRTI), while 13% were on a combination including a PI. The prevalence of raised TG was 10.5% (95% CI 7.5–13.9), overall; 13.2% (95% CI 8.1–19.2) among individuals on ART and 8.4% (95% CI 4.9–12.6) among individuals not on ART.

**Table 3.** Number of individuals (with data on triglycerides) receiving specific antiretroviral therapy drug class combination and the most common regimen in pooled analyses of the association between ant-retroviral therapy and cardiometabolic risk in sub-Saharan Africa

	Study								Total
	Sani	Dave	Pefura	GPC	Stehouwer	DDS	Kruger-Fourie	Walsh	
Number on ART	100	551	204	208	191	192	61	45	1552
Number for which ART regimen data are available (% of number on ART)	100 (100)	549 (99.6)	204 (100)	208 (100)	186 (97.4)	166 (86.5)	0 (0)	0 (0)	1413 (91)
Two NRTIs + one NNRTI (%)^a^	99 (99)	445 (81)	138 (68)	201 (97)	119 (64)	128 (77)	_	_	1130 (80)
One NRTI + one NNRTI (%)^a^						13 (8)	_	_	13 (1)
Two NRTIs + one PI^b^ (%)^a^	1 (1)	94 (17)	66 (32)	6 (3)	13 (17)	2 (1)	_	_	182 (13)
One NRTIs + one PI (%)^a^						2 (1)	_	_	2 (0)
One or two NNRTI only (%)^a^						2 (1)	_	_	2 (0)
One, two or three NRTIs only (%)^a^				1 (0)	54 (29)	19 (11)	_	_	74 (5)
One NRTI + one NNRTI + one PI (%)^a^		1 (0)					_	_	1 (0)
One NNRTI + one PI (%)^a^		9 (2)					_	_	9 (0)
Most common regimen	D4T or AZT/3TC/NVP	D4T or AZT/3TC/EFV; D4T or AZT/3TC/NVP	D4T or AZT/3TC/EFV; D4T or AZT/3TC/NVP	AZT/3TC/NVP	TDF, or D4T, or AZT/3TC/EFV; TDF, or D4T, or AZT/3TC/NVP	TDF/FTC/EFV	_	_	
Number on most common regimen (%)^c^	73 (74)	437 (98)	138 (100)	172 (86)	119 (100)	120 (94)	_	_	

ART, antiretroviral therapy; NRTI, nucleoside reverse transcriptase inhibitors; NNRTI, non-nucleoside reverse transcriptase inhibitors; PI, protease inhibitors; GPC, General Population Cohort; DDS, Durban Diabetes Study; D4T, stavudine; AZT, zidovudine; 3TC, lamivudine; NVP, niverapine; EFV, efavirenz; TDF, tenofovir; FTC, emtricitabine.

a Percentage of number for which ART regimen data are available.

b The PI was lopinavir/ritonavir, 80% of the time.

c Percentage of number receiving 2NRTIs + 1NNRTI.

^_^ Regimen data not provided.

Some non-zero proportions are recorded as 0% and some percentages do not add up to 100%, because of rounding errors.

### Association between ART and raised TG

**Fig. 2.**
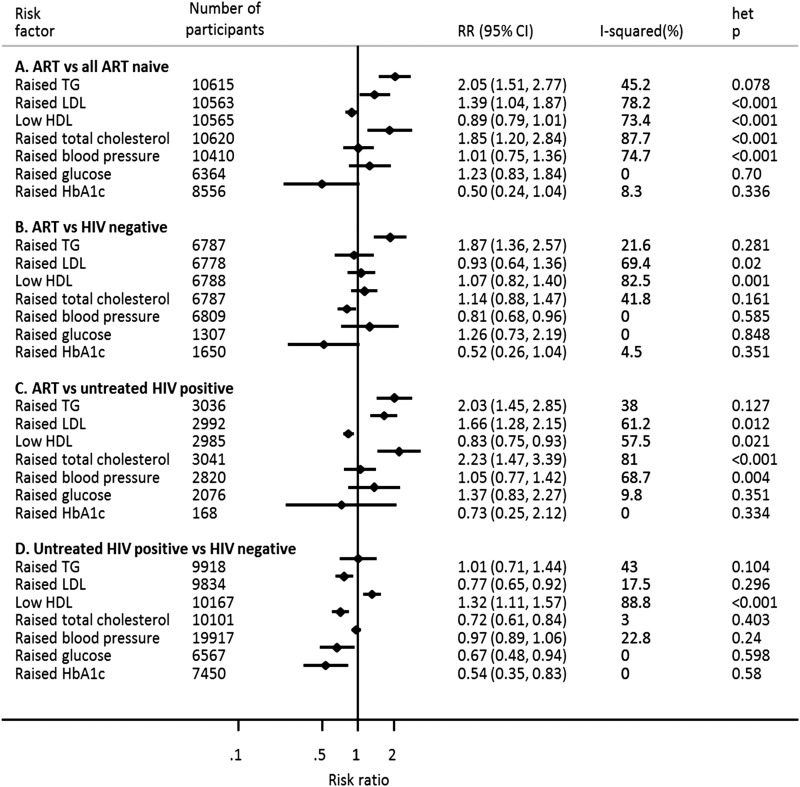
Association of anti-retroviral therapy and untreated HIV infection with selected cardiometabolic risk factors in sub-Saharan Africa.

Compared with individuals not receiving ART (i.e. untreated HIV-positive individuals only, or untreated HIV-positive individuals and HIV negative individuals combined), individuals receiving ART were two times more likely to have raised TG (RR 2.05, 95% CI 1.51–2.77) (Fig. 2*a*). This association did not vary substantially across studies (*I*^2^ 45.2%) and was consistent when ART users were compared with HIV-negative individuals only and with untreated HIV-positive individuals only (Fig. 2*b*, *c*). Additional analyses comparing untreated HIV-positive individuals with HIV-negative individuals found no association between untreated HIV infection and TG (Fig. 2*d*) suggesting that the association between ART and raised TG is independent of HIV infection.

### Association between ART drug class and raised TG

Additionally, we performed sensitivity analyses to assess whether there was a difference in probability of raised TG between individuals receiving an ART combination that included a PI and individuals receiving a combination not including a PI. In one of three studies with adequate numbers of individuals on PIs to allow analyses, we found some indication that individuals receiving a PI combination are more likely to have raised TG compared with individuals on a non-PI combination (Dave study: RR 2.10, 95% CI 1.06–4.02). However, this association was confounded by duration of ART. Individuals on ART drug combinations that included a PI had been on ART, on average, twice as long as individuals on non-PI combinations. Thus, when duration of ART was adjusted for, the association became non-significant (RR 1.5, 95% CI 0.72–3.11). There was no difference in the significance of the effect of PIs relative to non-PIs with and without adjustment for duration of ART in the other studies. It was, however, not possible to establish the time spent on a particular class of ART as the duration reported was simply time spent on all ART. Considering PIs are currently used as second-line drugs, individuals on PIs are likely to have spent some time on first-line non-PI combinations. Thus, in these data, there was no evidence that PIs were more strongly associated with raised TG than NNRTIs after adjusting for treatment duration. Additionally, the probability of raised TG was not significantly different between individuals whose regimen included AZT compared with stavudine and EFV compared with NVP.

### Association between ART and other cardiometabolic risk factors

The estimates of association of ART (in the primary analyses) with raised LDL (RR 1.39, 95% CI 1.04–1.87) and raised TC (RR 1.85, 95% CI 1.20–2.84), though significant, were markedly heterogeneous (*I*^2^ 78.2% and 87.7%, respectively) across studies (Fig. 2*a*). There was no evidence of an association between ART and raised BP, glucose, HbA1c or low HDL (Fig. 2*a*).

When ART users were compared separately with HIV-negative individuals and with untreated HIV-positive individuals, the association of ART with raised LDL, low HDL and raised TC was inconsistent across studies (Fig. 2*b*,*c*). There was no association between ART and raised glucose or HbA1c when comparing individuals on ART with HIV-negative individuals or untreated HIV-positive individuals (Fig. 2*b*,*c*). Additionally, ART was associated with a lower risk of raised BP when individuals on ART were compared with HIV-negative individuals (Fig. 2*b*). However, there was no evidence of association between ART and raised high BP in comparison with untreated HIV-positive individuals (Fig. 2*c*).

### Sources of heterogeneity and individual study influence on pooled association between ART and cardiometabolic risk factors

In primary analyses comparing individuals receiving ART to all other individuals, the magnitude of association between ART and raised TG ranged from 0.81 to 6.17, with a moderate level of heterogeneity (*I*^2^ = 45.2%) (Supplementary Fig. S1). We found no statistically significant study-level determinants of between-study heterogeneity in the pooled association between ART and TG (Supplementary Table S1). Additionally, no single study substantially influenced the pooled estimate of the association between ART and raised TG (Supplementary Table S2). In comparisons of individuals receiving ART with HIV-negative individuals, we found only minimal between-study heterogeneity in the association between ART and raised TG (*I*^2^ = 21.6%) (Supplementary Fig. S2). This heterogeneity was not explained by any of the study-level variables assessed (Supplementary Table S3), and the pooled association was not substantially influenced by any particular study (Supplementary Table S4). Similarly, in comparison of ART users with untreated HIV-positive individuals, between-study heterogeneity in the association between ART and raised TG was low (*I*^2^ = 38.0%) (Supplementary Fig. S3) and not associated with any of the study-level characteristics (Supplementary Table S5). The pooled RR was also not influenced by any one study (Supplementary Table S6). In comparisons of untreated HIV-positive individuals with HIV-negative individuals, as discussed above, we found no association between untreated HIV and raised TG. This lack of association was consistent across studies (*I*^2^ = 43.0%) (Supplementary Fig. S4) and was not significantly influenced by any of the study characteristics assessed (Supplementary Table S7). Lastly, the pooled magnitude of association was not influenced by a single study (Supplementary Table S8).

As indicated above, there was significant between-study heterogeneity in the association between ART and all the other cardiometabolic risk factors in the primary analysis, except for raised blood glucose and HbA1c. The observed heterogeneity was not associated with any of the study-level factors assessed, nor was the pooled measure of association influenced by one study alone, except for raised BP where the magnitude of association with ART tended to be higher in population studies compared with clinic-based studies (Supplementary Tables S1 and S2).

## Discussion

In these pooled analyses of 10 615 individuals, ART was independently associated with a twofold higher probability of raised TG. This could have important implications for the burden of CVD in SSA as access to, and duration on, ART increases in this population, highlighting the need to better understand the effect of long-term ART on TG and its impact on the burden of CVD in SSA.

Our findings are broadly consistent with earlier reports, including a study among black African women in rural South Africa and a meta-analysis of clinical trials in European-descent populations [31, 32]. These studies suggested that first-line ART is associated with raised cholesterol and TG [31, 32]. In addition, a study of metabolic complications in a European-descent population found that the use of combined NNRTI and PI was associated with a fivefold higher prevalence of hypertriglyceridaemia [33]. In our study, 87% of the participants on ART were either on two NRTIs and one NNRTI (the standard first-line anti-HIV drugs recommended across SSA) or a non-standard combination of NRTIs and NNRTIs; while only 13% received drug combinations including a PI [34]. PIs, currently used as second-line ART drugs, have been the most cited in studies measuring CVD among HIV-infected people receiving ART in other parts of the world [7, 33]. In this study, we found no evidence that ART combinations including PIs were more strongly associated with raised TG than non-PI combinations.

Many mechanisms by which ART may lead to raised TG levels have been proposed. It is thought that ART may reduce the clearance of TG from circulation through impairment of lipoprotein lipase activity in experimental studies [35]. Additionally, ART may cause accumulation of the sterol-sensing transcription factor SREBP, the chief regulator of lipid homeostasis, which contributes to an increase in hepatic intracellular lipids [36]. Further, ART may increase the level of circulating TG by altering mitochondrial proliferation, morphology and mitochondrial DNA content, or inhibiting the degradation of and increasing hepatic secretion of ApoB, the main lipoprotein for transportation of lipids [37–41].

Evidence of the effect of prolonged ART use on CVD risk is currently limited to a few studies in western populations. One study found a relative rate of myocardial infarction of between 0.98 and 1.13 per year of NNRTI exposure and 1.10 and 1.23 per year of PI exposure [7]. Similar to this study, our findings suggest that longer duration of ART may confer greater risk of raised TG. However, the potential impact of ART on CVD risk mediated specifically through raised TG may be inferred from studies of the effect of TG on CVD. For example, in a recent meta-analysis, the odds of coronary heart disease (CHD) was nearly doubled in individuals with TG values in the top third of the population compared with those in the bottom third [2]. Additionally, in another study, an increase of 1 mmol/L in TG was associated with increases of between 14% and 37% in CVD risk after adjustment for HDL [1]. Extrapolating the results of the studies above to our study, with TG higher in ART users by 1.11 mmol/L than the rest of the population on average, ART may be associated with 16–41% increase in CVD risk.

We note, however, that there is a paucity of published population data on the prevalence of dyslipidaemia, including raised TG, in SSA. Estimates of raised TG prevalence ranging from 5% to 20% have been reported in rural East Africa and urban West Africa, respectively [42–44]. This variation likely reflects differences in study design as well as potential real differences between populations. Further, available evidence consistently shows higher rates of hyperglyceridaemia among HIV-infected individuals receiving ART compared with untreated HIV-positive individuals and HIV-negative individuals [43, 45, 46]. Estimates of the prevalence of hypertriglyceridemia of between 14% and 42% have been reported among individuals receiving ART [43, 45, 46]. The heterogeneity is perhaps explained by underlying differences between populations in addition to differences in the duration of ART use – for example, comorbidities, socio-economic factors and healthcare systems.

The strength of this study is that it is the largest to date to assess the association between ART and cardiometabolic risk in SSA using IPD. In addition, we defined cardiometabolic risk factors according to clinically relevant cut-offs. Our findings may therefore be relevant for the clinical care of patients. Further, use of IPD enabled a more comprehensive adjustment for potential confounders including BMI and socio-demographic factors, as well as behavioural risk factors at the individual level. Importantly, the study assessed data on the class of ART thereby shedding more light on the impact of ART drug class on lipids, which is relevant to HIV patient treatment and care.

However, the study has some limitations. First, the majority of the studies included in the pooled analysis were cross-sectional. This precludes an analysis of the temporal relationship between ART use and the cardiometabolic risk factors studied. Second, our results may have been confounded by fasting status, as some of the studies included in the pooled analyses provided non-fasted lipids and glucose measurements. However, studies of fasting participants and studies of unfasted participants have reported only minor differences in the strength of associations between TG and CHD [2]. This, and the fact that non-fasted lipids and glucose were presented by only two studies, suggests that the impact of potential confounding due to differences in fasting status on the validity of our results is likely to be minimal.

In summary, this study provides evidence of association between ART and raised TG in SSA. Given the increasing use of ART and a potentially causal association between raised TG and CVD outcomes, our findings support the need to transition to new ARV drugs, such as dolutegravir, that have shown less adverse effects on lipids [47]. Importantly, the findings highlight the need for prospective studies to clarify the impact of long-term ART and its interplay with other risk factors on CVD risk in SSA. In the interim, it might be beneficial to strengthen the monitoring of lipid levels in individuals receiving ART.

## Supplementary material

For supplementary material accompanying this paper visit https://doi.org/10.1017/gheg.2018.7. click here to view supplementary material
