# Supplementary material for: HIV treatment is associated with a twofold higher probability of raised triglycerides: pooled analyses in 21 023 individuals in sub-Saharan Africa
Source: Glob Health Epidemiol Genom. 2018 May 8;3:e7. doi: 10.1017/gheg.2018.7 (PMC5985947; doi:10.1017/gheg.2018.7)
Supplement: Supplementary file 1 [file S2054420018000076sup.zip › S2054420018000076sup001.docx]

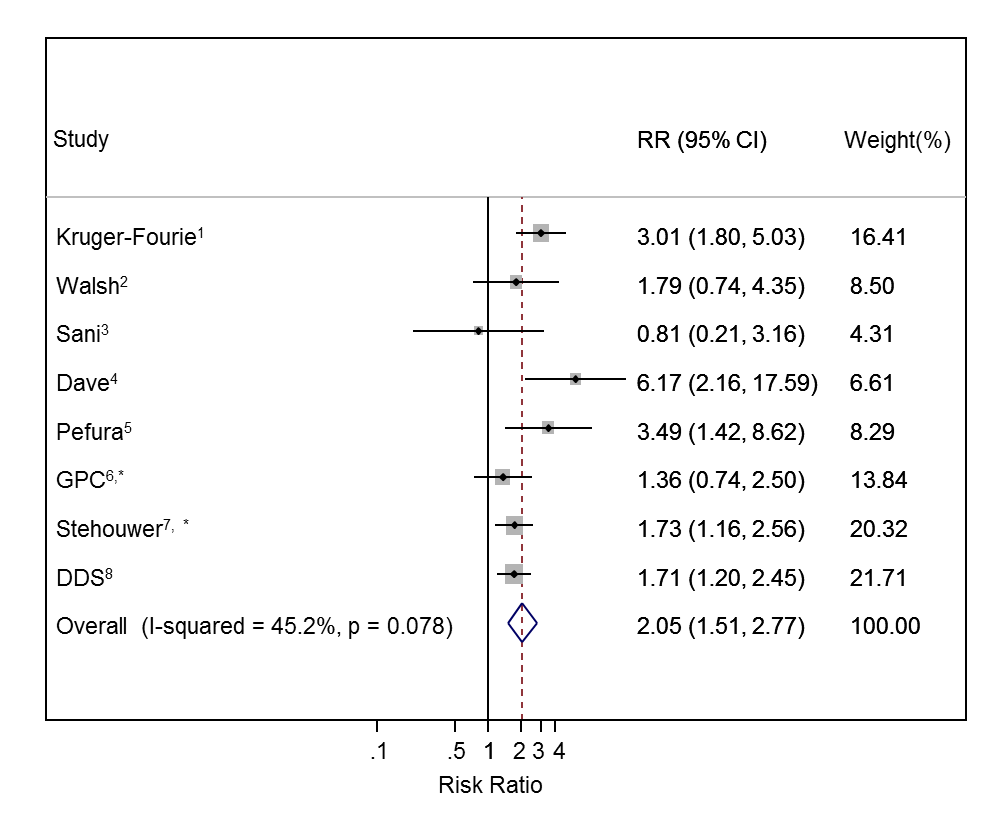


All studies adjusted for age, sex, body mass index, low-density lipoprotein, high-density lipoprotein and blood pressure. ^1^Also adjusted for alcohol, lipid medication, education, glucose and glycated haemoglobin; ^2^Also adjusted for smoking, alcohol, education, glucose and glycated haemoglobin; ^3^Also adjusted smoking, alcohol, lipid medication, education and glucose; ^4,5^Also adjusted for smoking, alcohol, education and glucose; ^6^Also adjusted for smoking, alcohol, diet, physical activity, cholesterol treatment, socio-economic position and glycated haemoglobin; ^7^Made no further adjustments; ^8^Also adjusted for smoking, alcohol, physical activity, occupation, education, socio-economic position and glucose; *Nonfasting samples; RR=Risk ratio comparing antiretroviral therapy users to antiretroviral therapy non-users.

**Fig S1. Association between antiretroviral therapy and raised triglycerides with individuals not on antiretroviral therapy (untreated HIV positive or untreated HIV positive and HIV negative combined) as the reference group**
