# Supplementary material for: HIV treatment is associated with a twofold higher probability of raised triglycerides: pooled analyses in 21 023 individuals in sub-Saharan Africa
Source: Glob Health Epidemiol Genom. 2018 May 8;3:e7. doi: 10.1017/gheg.2018.7 (PMC5985947; doi:10.1017/gheg.2018.7)
Supplement: Supplementary file 1 [file S2054420018000076sup.zip › S2054420018000076sup002.docx]

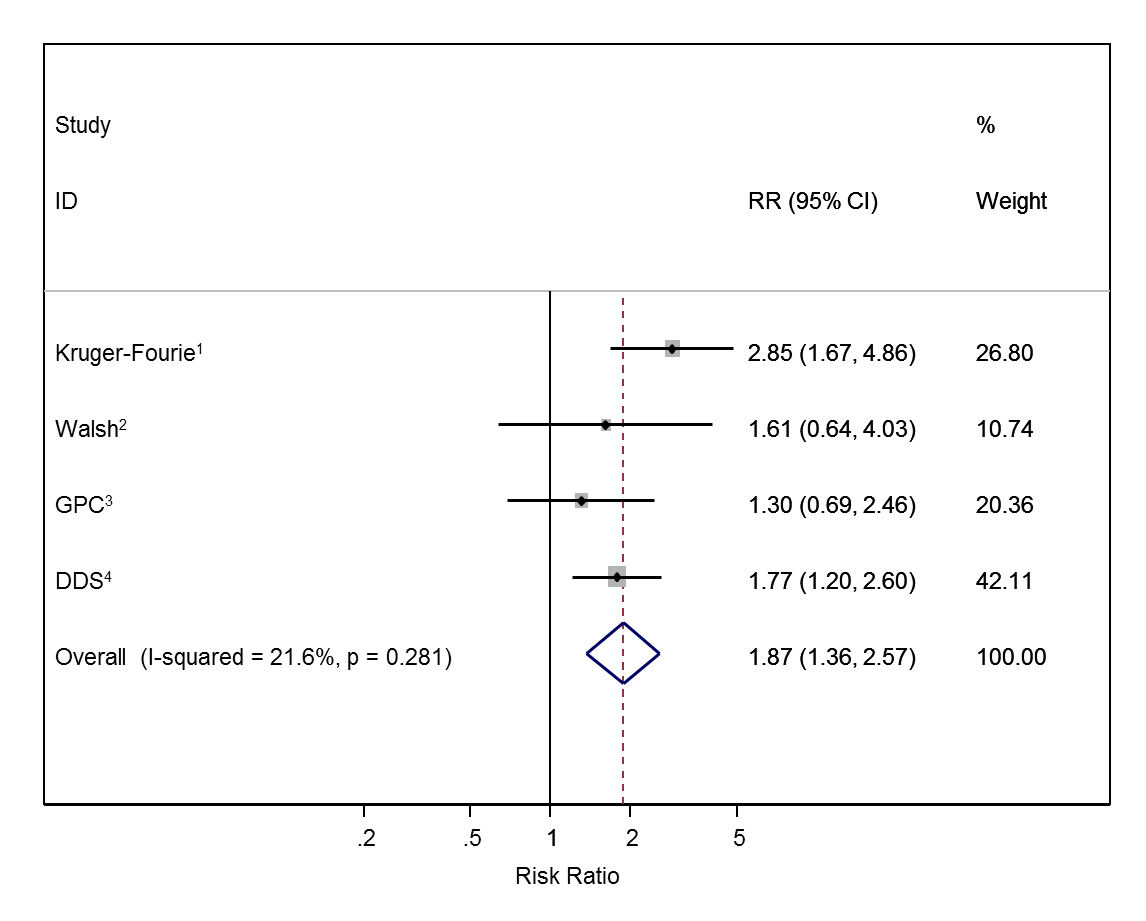


**Fig S2. Association between antiretroviral therapy and raised triglycerides with HIV negative individuals as the reference group**

All studies adjusted for age, sex, body mass index, low-density lipoprotein, high-density lipoprotein, blood pressure and glycated haemoglobin. ^1^Also adjusted for alcohol, lipid medication, education and glucose; ^2^Also adjusted for smoking, alcohol, education and glucose; ^3^Also adjusted for smoking, alcohol, diet, physical activity, cholesterol treatment and socio-economic position; ^4^Also adjusted for smoking, alcohol, physical activity, occupation, education, socio-economic position and glucose; RR=Risk Ratio comparing antiretroviral therapy users with HIV negative individuals
