# Supplementary material for: HIV treatment is associated with a twofold higher probability of raised triglycerides: pooled analyses in 21 023 individuals in sub-Saharan Africa
Source: Glob Health Epidemiol Genom. 2018 May 8;3:e7. doi: 10.1017/gheg.2018.7 (PMC5985947; doi:10.1017/gheg.2018.7)
Supplement: Supplementary file 1 [file S2054420018000076sup.zip › S2054420018000076sup003.docx]

**
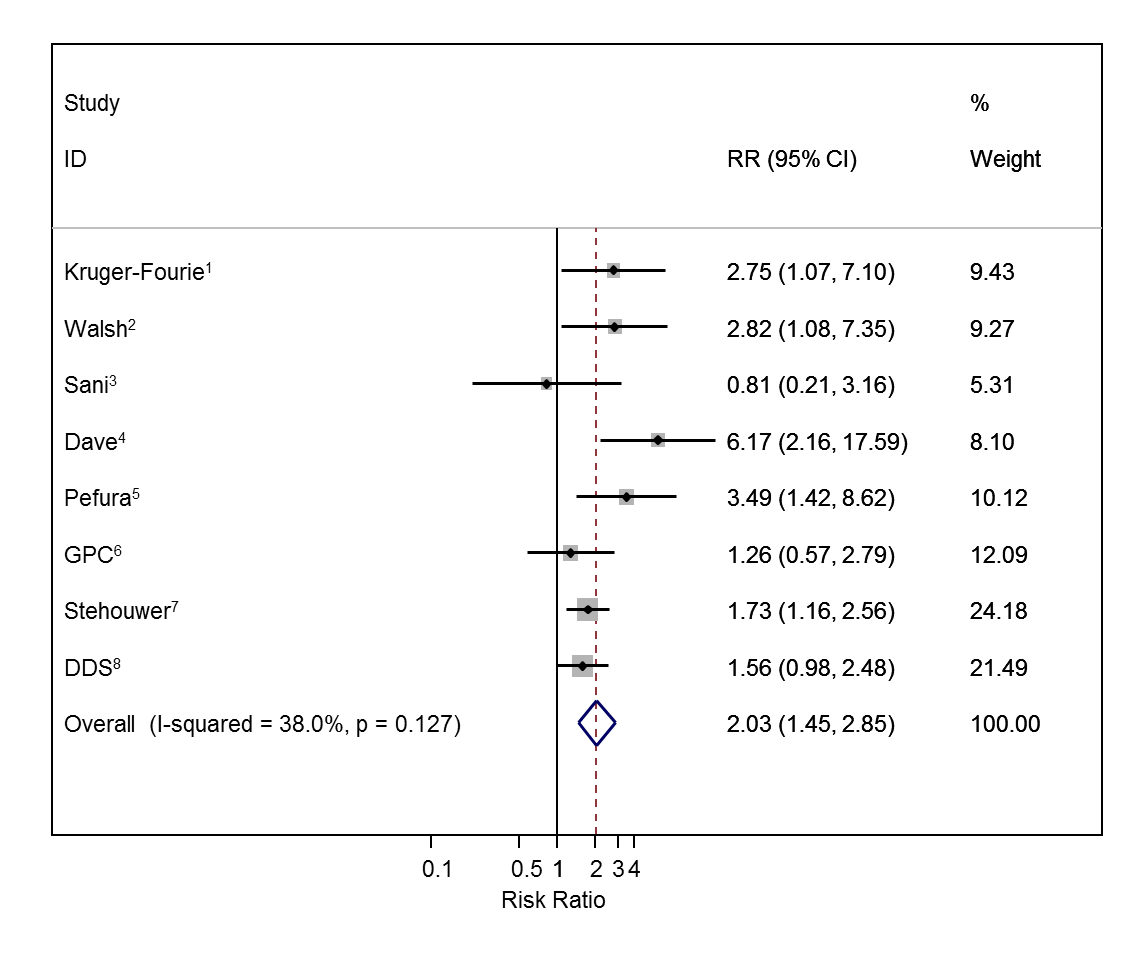
**

**Fig S3. Association between antiretroviral therapy and raised triglycerides with untreated HIV positive individuals as the reference group**

All studies adjusted for age, sex and body mass index; ^1^Also adjusted for low-density lipoprotein, high-density lipoprotein, blood pressure, alcohol, lipid medication, education, glucose and glycated haemoglobin; ^2^Also adjusted for low-density lipoprotein, high-density lipoprotein, blood pressure, smoking, alcohol, education glucose and glycated haemoglobin; ^3^Also adjusted for low-density lipoprotein, high-density lipoprotein, blood pressure, smoking, alcohol, lipid medication, education and glucose; ^4,5^Also adjusted for low-density lipoprotein, high-density lipoprotein, blood pressure, smoking, alcohol, education and glucose; ^6^Also adjusted for low-density lipoprotein, high-density lipoprotein, blood pressure, smoking, alcohol, diet, physical activity, cholesterol treatment, socio-economic position and glycated haemoglobin; ^7^Made no further adjustments; ^8^Also adjusted for low-density lipoprotein, high-density lipoprotein, blood pressure, physical activity, occupation, education, socio-economic position, glucose and glycated haemoglobin; RR=Risk Ratio comparing antiretroviral therapy users with untreated HIV positive individuals.
