# Supplementary material for: HIV treatment is associated with a twofold higher probability of raised triglycerides: pooled analyses in 21 023 individuals in sub-Saharan Africa
Source: Glob Health Epidemiol Genom. 2018 May 8;3:e7. doi: 10.1017/gheg.2018.7 (PMC5985947; doi:10.1017/gheg.2018.7)
Supplement: Supplementary file 1 [file S2054420018000076sup.zip › S2054420018000076sup005.docx]

**Table S1. Results of meta-regression assessing possible sources of heterogeneity in pooled analyses of the association between anti-retroviral therapy (ART)^‡^ and selected cardiometabolic risk factors in sub-Saharan Africa**

|  | Raised TG | |  | Raised LDL | |  | Low HDL | |  | Raised TC | |  | Raised BP | |
| --- | --- | --- | --- | --- | --- | --- | --- | --- | --- | --- | --- | --- | --- | --- |
| Number of studies | 8 | |  | 8 | |  | 8 | |  | 8 | |  | 7 | |
| Unadjusted *I^2^ %* | 45.2 | |  | 78.2 | |  | 73.4 | |  | 87.7 | |  | 74.7 | |
| Explanatory variable | ^^^β(95%CI) | % of *Ʈ*^2^ explained* |  | ^^^β(95%CI) | % of *Ʈ*^2^ explained* |  | ^^^β(95%CI) | % of *Ʈ*^2^ explained* |  | ^^^β(95%CI) | % of *Ʈ*^2^ explained* |  | ^^^β(95%CI) | % of *Ʈ*^2^ explained* |
| Study type | 0.03 (-0.68-0.75) | -72.23 |  | 0.31 (-0.24-0.87) | 11.06 |  | -0.10(-0.36-0.16) | -3.26 |  | 0.21 (-0.71-1.15) | -15.39 |  | 0.73(0.17-1.29) | 100 |
| Study size | 0.00 (-0.00-0.00) | -17.13 |  | 0.00 (0.00-0.00) | 27.69 |  | 0.00 (0.00-0.00) | -19.35 |  | 0.00 (0.00-0.00) | -7.25 |  | 0.00(0.00-0.00) | 78.79 |
| Year of study | -0.01(-0.16-0.14) | -76.02 |  | -0.03 (-0.17-0.12) | -21.84 |  | 0.00 (-0.06-0.06) | -25.14 |  | -0.08(-0.29-0.13) | -5.19 |  | -0.07(-0.28-0.15) | -91.81 |
| Location | -0.17 (-0.79-0.45) | -51.48 |  | 0.10 (-0.44-0.64) | -21.53 |  | -0.12 (-0.32-0.08) | 15.36 |  | 0.21 (-0.59-1.02) | -14.14 |  | 0.65(-0.07-1.36) | 36.22 |
| Proportion of males | -0.03 (-0.09-0.03) | -47.99 |  | 0.00 (-0.06-0.05) | -23.86 |  | -0.01 (-0.03-0.01) | 14.79 |  | 0.01 (-0.07-0.09) | -19.01 |  | 0.03(-0.04-0.11) | -75.07 |
| Mean BMI | 0.04 (-0.17-0.24) | -111.97 |  | 0.00 (-0.21-0.21) | 25.45 |  | 0.05 (-0.03-0.2) | 21.54 |  | -0.05 (-0.36-0.25) | -18.76 |  | 0-.08(-0.37-0.21) | -80.07 |
| Mean Age | 0.03 (-0.03-0.08) | 100 |  | -0.01 (-0.08-0.06) | -25.3 |  | 0.02 (0.00-0.04) | 75 |  | -0.06 (-0.14-0.03) | 21.21 |  | -0.04(-0.11-0.03) | 29.25 |
| β = meta-regression coefficient; CI = Confidence Interval; Ʈ2 = between study heterogeneity; ^ β=0.00 and 95% CI = (0.00 - 0.00) is due to rounding errors; TG=Triglycerides, LDL=Low density lipoprotein, HDL=High density lipoprotein, TC=Total cholesterol, BP=Blood pressure, BMI=Body mass index; *Negative means the potential explanatory variable increases heterogeneity; ‡ART users are compared with non-users (untreated HIV positive or untreated HIV positive and HIV negative individuals combined). | | | | | | | | | | | | | | |
