# Supplementary material for: HIV treatment is associated with a twofold higher probability of raised triglycerides: pooled analyses in 21 023 individuals in sub-Saharan Africa
Source: Glob Health Epidemiol Genom. 2018 May 8;3:e7. doi: 10.1017/gheg.2018.7 (PMC5985947; doi:10.1017/gheg.2018.7)
Supplement: Supplementary file 1 [file S2054420018000076sup.zip › S2054420018000076sup006.docx]

**Table S2. Sensitivity analysis assessing the influence of a single study on the pooled risk ratio comparing ART users to non-users in pooled analyses of association between ART and selected cardiometabolic risk factors in sub-Saharan Africa**

| Study omitted | Pooled RR(95% CI) | Pooled RR(95% CI) | Pooled RR(95% CI) | Pooled RR(95% CI) | Pooled RR(95% CI) | Pooled RR(95% CI) | Pooled RR(95% CI) |
| --- | --- | --- | --- | --- | --- | --- | --- |
|  | Raised TG | Raised LDL | Raised HDL | Raised TC | Raised BP | Raised Glucose | Raised HbA1c |
| No study excluded | 2.05(1.51-2.77) | 1.39(1.04-1.87) | 0.89(0.79-1.01) | 1.85(1.20-2.84) | 1.01(0.75-1.36) | 1.26(0.84-1.90) | 0.5(0.24-1.04) |
| Kruger-Fourie | 1.88(1.38-2.58) | 1.36(0.95-1.95) | 0.85(0.77-0.94) | 1.93(1.15-3.26) | 1.10(0.80-1.50) | _ | 0.61(0.31-1.20) |
| Walsh | 2.09(1.49-2.92) | 1.50(1.13-2.01) | 0.88(0.77-1.00) | 2.10(1.35-3.25) | 1.05(0.72-1.52) | _ | 0.43(0.12-1.60) |
| Sani | 2.13(1.57-2.88) | 1.42(1.03-1.98) | 0.91(0.79-1.05) | 1.69(1.08-2.64) | 0.95(0.74-1.21) |  |  |
| Dave | 1.89(1.46-2.43) | 1.30(0.95-1.78) | 0.91(0.781-1.07) | 1.62(1.08-2.42) | 1.02(0.73-1.44) | 1.42(0.91-2.23) |  |
| Pefura | 1.95(1.42-2.67) | 1.31(0.95-1.80) | 0.89(0.78-1.03) | 1.94(1.19-3.17) | _ |  |  |
| GPC | 2.19(1.57-3.06) | 1.50(1.12-2.02) | 0.91(0.79-1.05) | 1.97(1.22-3.18) | 1.07(0.75-1.52) | _ | _ |
| Stehouwer | 2.15(1.47-3.15) | 1.29(0.95-1.75) | 0.92(0.80-1.06) | 1.62(1.08-2.44) | 0.90(0.69-1.17) | 1.26(0.79-2.03) |  |
| DDS | 2.17(1.48-3.18) | 1.46(1.07-2.01) | 0.87(0.76-0.98) | 1.96(1.16-3.30) | 1.06(0.75-1.50) | 1.18(0.72-1.94) | 0.22(0.05-0.89) |
| Faurholt-Jepsen | _ | _ | _ | _ | _ | 1.16(0.73-1.87) | _ |
| TG=Triglycerides; LDL=Low density lipoprotein cholesterol; HDL=High density lipoprotein cholesterol; TC=Total Cholesterol; HbA1c=Glycated haemoglobin; ART=Antiretroviral therapy; CI =Confidence Interval; _ study did not have relevant data | | | | | | | |
