# Supplementary material for: HIV treatment is associated with a twofold higher probability of raised triglycerides: pooled analyses in 21 023 individuals in sub-Saharan Africa
Source: Glob Health Epidemiol Genom. 2018 May 8;3:e7. doi: 10.1017/gheg.2018.7 (PMC5985947; doi:10.1017/gheg.2018.7)
Supplement: Supplementary file 1 [file S2054420018000076sup.zip › S2054420018000076sup007.docx]

**Table S3: Results of meta-regression assessing possible sources of heterogeneity in pooled analyses of association between anti-retroviral therapy (ART)^‡^ and selected cardiometabolic risk factors in Sub Saharan Africa**

|  | Raised TG | |  | Raised LDL | |  | Low HDL | |  | Raised TC | |  |
| --- | --- | --- | --- | --- | --- | --- | --- | --- | --- | --- | --- | --- |
| Number of studies | 4 | |  | 4 | |  | 4 | |  | 4 | |  |
| Unadjusted *I^2^ %* | 21.6 | |  | 69.4 | |  | 82.5 | |  | 41.8 | |  |
| Explanatory variable | ^^^β(95%CI) | % of *Ʈ*^2^ explained* |  | ^^^β(95%CI) | % of *Ʈ*^2^ explained* |  | ^^^β(95%CI) | % of *Ʈ*^2^ explained* |  | ^^^β(95%CI) | % of *Ʈ*^2^ explained* |  |
| Study type | # | # |  | # | # |  | # | # |  | # | # |  |
| Study size | 0.00(0.00-0.00) | 0 |  | 0.00 (0.00-0.00) | -0.32 |  | 0.00 (0.00-0.00) | 72.68 |  | 0.00 (0.00-0.00) | 0 |  |
| Year of study | 0.05 (-0.25-0.00) | 0 |  | -0.05 (-0.34-0.23) | -48.3 |  | -0.05 (-0.24-0.15) | -5.58 |  | -0.02(-0.30-0.27) | 0 |  |
| Location | -0.40 (-1.96-1.16) | 0 |  | -0.48 (-2.79-1.84) | 12.04 |  | -0.40 (-1.19-0.40) | 73.53 |  | 0.13 (-2.15-2.42) | 0 |  |
| Proportion of males | -0.01 (-0.13-0.11) | 0 |  | 0.01 (-0.15-0.16) | -76.89 |  | -0.02 (-0.09-0.05) | 28.79 |  | 0.03 (-0.08-0.14) | 0 |  |
| Mean BMI | 0.03 (-0.27-0.33) | 0 |  | 0.04 (-0.37-0.44) | -75.55 |  | 0.04 (-0.17-0.26) | -15.39 |  | -0.02 (-0.38-0.35) | 0 |  |
| Mean Age | 0.03 (-0.07-0.14) | 0 |  | 0.03 (-0.11-0.17) | -22.29 |  | 0.03 (-0.04-0.11) | 65.39 |  | 0.00 (-0.15-0.16) | 0 |  |
| β = meta-regression coefficient; CI = Confidence Interval; Ʈ2 = between study heterogeneity; ^ β=0.00 and 95% CI = (0.00 - 0.00) is due to rounding errors; TG=Triglycerides, LDL=Low density lipoprotein, HDL=High density lipoprotein, TC=Total cholesterol, BP=Blood pressure, BMI=Body mass index; # All studies are population based; *Negative means the potential explanatory variable explain less of the heterogeneity than would be expected by chance. **^‡^**Comparisons are between ART users and HIV negative individuals | | | | | | | | | | | | |
