# Supplementary material for: HIV treatment is associated with a twofold higher probability of raised triglycerides: pooled analyses in 21 023 individuals in sub-Saharan Africa
Source: Glob Health Epidemiol Genom. 2018 May 8;3:e7. doi: 10.1017/gheg.2018.7 (PMC5985947; doi:10.1017/gheg.2018.7)
Supplement: Supplementary file 1 [file S2054420018000076sup.zip › S2054420018000076sup008.docx]

**Table S4: Sensitivity analysis assessing the influence of a single study on the pooled risk ratio comparing ART users to HIV negative individuals in pooled analyses of association between antiretroviral therapy and selected cardiometabolic risk factors in Sub Saharan Africa**

| Study omitted | Pooled RR(95% CI) | Pooled RR(95% CI) | Pooled RR(95% CI) | Pooled RR(95% CI) | Pooled RR(95% CI) | Pooled RR(95% CI) | Pooled RR(95% CI) |
| --- | --- | --- | --- | --- | --- | --- | --- |
|  | Raised TG | Raised LDL | Raised HDL | Raised TC | Raised BP | Raised Glucose | Raised HbA1c |
| No study excluded | 1.87(1.36-2.57) | 0.93(0.64-1.36) | 1.07(0.82-1.40) | 1.14(0.88-1.47) | 0.81(0.68-0.96) | 1.26(0.73-2.19) | 0.52(0.26-1.04) |
| Kruger-Fourie | 1.63(1.19-2.22) | 0.78(0.53-1.17) | 0.97(0.75-1.24) | 1.02(0.67-1.54) | 0.86(0.71-1.05) | _ | 0.62(0.31-1.22) |
| Walsh | 1.90(1.27-2.83) | 1.04(0.71-1.51) | 1.03(0.73-1.45) | 1.24(1.03-1.49) | 0.77(0.63-0.95) | _ | 0.39(0.11-1.41) |
| GPC | 2.04(1.47-2.83) | 1.03(0.69-1.53) | 1.20(0.95-1.51) | 1.09(0.76-1.55) | 0.80(0.65-0.98) | _ | _ |
| DDS | 1.90(1.12-3.21) | 0.84(0.43-1.64) | 1.10(0.73-1.66) | 1.03(0.65-1.62) | 0.80(0.66-0.97) | 1.19(0.52-2.72) | 0.34(0.06-2.02) |
| Faurholt-Jepsen | _ | _ | _ | _ | _ | 1.33(0.64-2.76) | _ |

TG=Triglycerides; LDL=Low density lipoprotein cholesterol; HDL=High density lipoprotein cholesterol; TC=Total cholesterol; HbA1c=Glycated haemoglobin; ART=Antiretroviral therapy; CI =Confidence Interval; GPC=General Population Cohort; DDS=Durban Diabetes Study; _ means study did not have relevant data
