# Supplementary material for: HIV treatment is associated with a twofold higher probability of raised triglycerides: pooled analyses in 21 023 individuals in sub-Saharan Africa
Source: Glob Health Epidemiol Genom. 2018 May 8;3:e7. doi: 10.1017/gheg.2018.7 (PMC5985947; doi:10.1017/gheg.2018.7)
Supplement: Supplementary file 1 [file S2054420018000076sup.zip › S2054420018000076sup009.docx]

**Table S5: Results of meta-regression assessing possible sources of heterogeneity in pooled analyses of association between anti-retroviral therapy (ART) and selected cardiometabolic risk factors (when compared with untreated HIV positive) in Sub Saharan Africa**

|  | Raised TG | |  | Raised LDL | |  | Low HDL | |  | Raised TC | |  | Raised BP | |
| --- | --- | --- | --- | --- | --- | --- | --- | --- | --- | --- | --- | --- | --- | --- |
| Number of studies | 8 | |  | 8 | |  | 8 | |  | 8 | |  | 7 | |
| Unadjusted *I^2^ %* | 38 | |  | 61.2 | |  | 57.5 | |  | 81 | |  | 68.7 | |
| Explanatory variable | ^^^β(95%CI) | % of *Ʈ*^2^ explained* |  | ^^^β(95%CI) | % of *Ʈ*^2^ explained* |  | ^^^β(95%CI) | % of *Ʈ*^2^ explained* |  | ^^^β(95%CI) | % of *Ʈ*^2^ explained* |  | ^^^β(95%CI) | % of *Ʈ*^2^ explained* |
| Study type | 0.04 (-0.70-0.78) | -112.76 |  | 0.15 (-0.32-0.62) | -18.29 |  | -0.04 (-0.28-0.20) | -25.35 |  | 0.03 (-0.73-0.79) | -23.29 |  | -1.08 (-2.62-0.47) | 6.76 |
| Study size | 0.00 (-0.00-0.00) | -113.75 |  | 0.00 (0.00-0.00) | -16.75 |  | 0.00 (0.00-0.00) | -37.94 |  | 0.00 (0.00-0.00) | 13.88 |  | 0.00 (0.00-0.00) | 100 |
| Year of study | 0.01 (-0.16-0.19) | -156.97 |  | -0.03 (-0.14-0.08) | -22.08 |  | 0.02 (0.03-0.07) | -3.06 |  | -0.09(-0.25-0.07) | 13.12 |  | 0.02 (-0.01-0.05) | 43.51 |
| Location | -0.18 (-0.84-0.48) | -131.08 |  | 0.00 (-0.41-0.41) | -31.78 |  | -0.07 (-0.6-0.12) | -9.61 |  | 0.07 (-0.60-0.74) | -22.06 |  | -0.07 (-0.47-0.32) | -20.47 |
| Proportion of males | -0.03 (-0.09-0.04) | -135.66 |  | 0.00 (-0.04-0.04) | -32.5 |  | -0.01 (-0.02-0.01) | -14.32 |  | 0.01 (-0.05-0.08) | -18.73 |  | 0.00 (-0.01-0.02) | -39.59 |
| Mean BMI | 0.03 (-0.21-0.26) | -180.59 |  | -0.01 (-0.18-0.16) | -30.13 |  | 0.05 (-0.02-0.11) | 27.09 |  | 0.01 (-0.25-0.28) | -22.93 |  | 0.01 (-0.05-0.07) | -40.3 |
| Mean Age | 0.03 (-0.10-0.17) | -26.75 |  | 0.03 (-0.04-0.10) | 5.28 |  | 0.00 (-0.03-0.04) | -27.95 |  | -0.05 (-0.17-0.08) | -6.71 |  | -0.01 (-0.03-0.00) | 100 |
|  |  |  |  |  |  |  |  |  |  |  |  |  |  |  |

TG=Triglycerides; LDL=Low density lipoprotein cholesterol; HDL=High density lipoprotein cholesterol; TC=Total cholesterol; HbA1c=Glycated haemoglobin; ART=Antiretroviral therapy; CI =Confidence Interval; GPC=General Population Cohort; DDS=Durban Diabetes Study; _ means study did not have relevant data
