# Supplementary material for: HIV treatment is associated with a twofold higher probability of raised triglycerides: pooled analyses in 21 023 individuals in sub-Saharan Africa
Source: Glob Health Epidemiol Genom. 2018 May 8;3:e7. doi: 10.1017/gheg.2018.7 (PMC5985947; doi:10.1017/gheg.2018.7)
Supplement: Supplementary file 1 [file S2054420018000076sup.zip › S2054420018000076sup010.docx]

**Table S6: Sensitivity analysis of change in pooled risk ratio comparing ART users with untreated HIV positive individuals, for each cardiometabolic risk factor**

| Study omitted | Pooled RR (95%CI) |  | Pooled RR (95%CI) |  | Pooled RR (95%CI) |
| --- | --- | --- | --- | --- | --- |
|  |  |  |  |  |  |
|  | Raised TG |  | Raised LDL |  | Raised HDL |
|  |  |  |  |  |  |
| No study excluded | 2.03(1.45-2.85) |  | 1.66(1.28-2.15) |  | 0.83(0 .74-0.93) |
| Dave | 1.79(1.39-2.30) |  | 1.58(1.17-2.12) |  | 0.84(0.73-0.97) |
| Kruger-Fourie | 1.98(1.37-2.88) |  | 1.58(1.19-2.11) |  | 0.82(0.73-0.92) |
| GPC | 2.19(1.51-3.17) |  | 1.70(1.30-2.24) |  | 0.85(0.76-0.95) |
| Sani | 2.13(1.52-3.00) |  | 1.76(1.34-2.31) |  | 0.84(0.74-0.95) |
| Walsh | 1.978(1.37-2.87) |  | 1.73(1.33-2.25) |  | 0.84(0.74-0.95) |
| Stehouwer | 2.18(1.40-3.39) |  | 1.56(1.18-2.07) |  | 0.85(0.76-0.96) |
| Pefura | 1.91(1.35-2.70) |  | 1.59(1.19-2.13) |  | 0.83(0.74-0.93) |
| DDS | 2.22(1.46-3.36) |  | 1.80(1.39-2.32) |  | 0.80(0.75-0.84) |
|  |  |  |  |  |  |
|  | Raised TC |  | Raised BP |  | Raised Glucose |
|  |  |  |  |  |  |
| No study excluded | 2.23(1.47-3.39) |  | 1.05(0.77-1.42) |  | 1.37(0.83-2.27) |
| Dave | 2.00(1.33-3.03) |  | 1.06(0.74-1.53) |  | 1.67(0.98-2.86) |
| Kruger-Fourie | 2.24(1.38-3.62) |  | 1.11(0.78-1.57) |  | _ |
| GPC | 2.31(1.46-3.66) |  | 1.10(0.78-1.55) |  | _ |
| Sani | 2.08(1.34-3.23) |  | 0.98(0.77-1.26) |  | 1.43(0.81-2.50) |
| Walsh | 2.43(1.57-3.76) |  | 1.08(0.74-1.59) |  | _ |
| Stehouwer | 2.01(1.32-3.04) |  | 0.94(0.70-1.25) |  | 1.41(0.67-2.94) |
| Pefura | 2.43(1.54-3.83) |  | _ |  | _ |
| Faurholt-Jepsen | _ |  | _ |  | 1.11(0.64-1.92) |
| DDS | 2.41(1.53-3.80) |  | 1.11(0.80-1.56) |  |  |

TG=Triglycerides; LDL=Low density lipoprotein cholesterol; HDL=High density lipoprotein cholesterol; TC=Total Cholesterol; BP=Blood pressure; ART=Antiretroviral therapy; CI =Confidence Interval; _ means study did not have relevant data; GPC is acronym for General Population Cohort; DDS is acronym for Durban Diabetes Study. Note: the other studies bear the name of the collaborator(s) contributing data for pooled analyses; HbA1c excluded because only two studies were included in this part of the analysis.
