# Supplementary material for: HIV treatment is associated with a twofold higher probability of raised triglycerides: pooled analyses in 21 023 individuals in sub-Saharan Africa
Source: Glob Health Epidemiol Genom. 2018 May 8;3:e7. doi: 10.1017/gheg.2018.7 (PMC5985947; doi:10.1017/gheg.2018.7)
Supplement: Supplementary file 1 [file S2054420018000076sup.zip › S2054420018000076sup011.docx]

**Table S7: Results of meta-regression assessing possible sources of heterogeneity in pooled analyses of association between untreated HIV infection and selected cardiometabolic risk factors (with HIV negative individuals as the reference group) in Sub Saharan Africa**

|  | Raised TG | |  | Raised LDL | |  | Low HDL | |  | Raised BP | |
| --- | --- | --- | --- | --- | --- | --- | --- | --- | --- | --- | --- |
| Number of studies | 7 | |  | 7 | |  | 7 | |  | 9 | |
| Unadjusted *I^2^ %* | 43 | |  | 17.5 | |  | 88.8 | |  | 22.8 | |
| Explanatory variable | ^^^β (95%CI) | % of *Ʈ*^2^ explained |  | ^^^β (95%CI) | % of *Ʈ*^2^ explained* |  | ^^^β (95%CI) | % of *Ʈ*^2^ explained* |  | ^^^β (95%CI) | % of *Ʈ*^2^ explained* |
| Study type | 1.57 (-0.40-3.55) | 0 |  | 0.58 (-1.44-2.60) | -8.5 |  | 0.32 (-0.43-1.06) | 4 |  | -1.08 (-2.62-0.45) | 6.8 |
| Study size | 0.00 (-0.00-0.00) | 0 |  | 0.00 (0.00-0.00) | -31.5 |  | 0.00 (0.00-0.00) | -13.3 |  | 0.00 (0.00-0.00) | 100 |
| Year of study | 0.05 (-0.10-0.19) | 0 |  | -0.01 (-0.08-0.07) | -101.9 |  | -0.04 (-0.12-0.04) | 5.5 |  | 0.02 (-0.01-0.05) | 43.5 |
| Location | 0.54 (-0.55-1.64) | 0 |  | -0.15 (-0.97-0.67) | -10.6 |  | 0.09 (-0.51-0.70) | -19.8 |  | -0.07 (-0.47-0.32) | -20.5 |
| Proportion of males | -0.02 (-0.07-0.02) | 0 |  | 0.00 (-0.02-0.03) | -25.2 |  | -0.01 (-0.02-0.01) | -3.9 |  | 0.00 (-0.01-0.02) | -39.6 |
| Mean BMI | -0.05 (-0.26-0.16) | 0 |  | 0.01 (-0.11-0.12) | -66 |  | -0.04 (-0.14-0.06) | 0.7 |  | 0.01 (-0.05-0.07) | -40.3 |
| Mean Age | -0.03 (-0.12-0.06) | 0 |  | -0.02 (-0.06-0.01) | 100 |  | 0.02 (-0.02-0.06) | 13.3 |  | -0.01 (-0.03-0.00) | 100 |
|  |  |  |  |  |  |  |  |  |  |  |  |
| β = meta-regression coefficient; CI = Confidence Interval; Ʈ2 = between study heterogeneity; ^ β=0.00 and 95% CI = (0.00 - 0.00) is due to rounding errors; TG=Triglycerides, LDL=Low density lipoprotein cholesterol, HDL=High density lipoprotein cholesterol, BP=Blood pressure, BMI=Body mass index; *Negative means the potential explanatory variable explains less of the heterogeneity than would be expected by chance | | | | | | | | | | | |
