# Supplementary material for: HIV treatment is associated with a twofold higher probability of raised triglycerides: pooled analyses in 21 023 individuals in sub-Saharan Africa
Source: Glob Health Epidemiol Genom. 2018 May 8;3:e7. doi: 10.1017/gheg.2018.7 (PMC5985947; doi:10.1017/gheg.2018.7)
Supplement: Supplementary file 1 [file S2054420018000076sup.zip › S2054420018000076sup012.docx]

**Table S8: Sensitivity analysis assessing the influence of a single study on the pooled risk ratio comparing untreated HIV positive to HIV negative individuals in pooled analyses of association between antiretroviral therapy and selected cardiometabolic risk factors in Sub Saharan Africa**

| Study omitted | Pooled RR(95% CI) |  | Pooled RR(95% CI) |  | Pooled RR(95% CI) |  | Pooled RR(95% CI) |
| --- | --- | --- | --- | --- | --- | --- | --- |
|  |  |  |  |  |  |  |  |
|  | Raised TG |  | Raised LDL |  | Raised HDL |  | Raised TC |
|  |  |  |  |  |  |  |  |
| No study excluded | 1.01(0.71-1.44) |  | 0.77(0.65-0.92) |  | 1.32(1.11-1.57) |  | 0.72(0.61-0.84) |
| Kruger-Fourie | 1.05(0.670-1.57) |  | 0.79(0.65-0.97) |  | 1.26(1.05-1.50) |  | 0.73(0.61-0.89) |
| GPC | 0.98(0.62-1.54) |  | 0.79(0.66-0.95) |  | 1.35(1.09-1.67) |  | 0.70(0.58-084) |
| Mutimura | 0.98(0.74-1.27) |  | 0.76(0.64-0.92) |  | 1.27(1.06-1.52) |  | 0.71(0.59-0.84) |
| Schutte | 0.99(0.67-1.48) |  | 0.76(0.62-0.93) |  | 1.30(1.08-1.57) |  | 0.70(0.58-0.84) |
| THUSA | 1.10(0.82-1.46) |  | 0.73(0.60-0.89) |  | 1.36(1.10-1.69) |  | 0.71(0.59-0.87) |
| Walsh | 1.07(0.70-1.63) |  | 0.83(0.71-0.98) |  | 1.28(1.06-1.55) |  | 0.77(0.65-0.91) |
| DDS | 0.98(0.62-1.56) |  | 0.73(0.59-0.89) |  | 1.41(1.21-1.65) |  | 0.65(0.54-0.79) |
|  |  |  |  |  |  |  |  |
|  | Raised BP |  | Raised Glucose |  | Raised HbA1c |  |  |
|  |  |  |  |  |  |  |  |
| No study excluded | 0.97(0.89-1.06) |  | 0.67(0.48-0.94) |  | 0.54(0.35-0.83) |  |  |
| Kruger-Fourie | 0.99(0.91-1.08) |  | 0.66(0.46-0.97) |  | 0.53(0.29-0.97) |  |  |
| GPC | 0.97(0.88-1.07) |  | _ |  | 0.50(0.32-0.79) |  |  |
| Mutimura | 0.98(0.91-1.06) |  | 0.69(0.49-0.97) |  |  |  |  |
| Schutte | 0.97(0.88-1.07) |  | 0.68(0.48-0.96) |  |  |  |  |
| THUSA | 0.97(0.87-1.07) |  | 0.63(0.44-0.90) |  |  |  |  |
| Walsh | 0.97(0.88-1.08) |  | 0.70(0.49-1.00) |  | 0.56(0.36-0.90) |  |  |
| Africa centre (2003) | 0.99(0.89-1.08) |  | _ |  | _ |  |  |
| Africa centre (2010) | 0.93(0.85-1.01) |  | _ |  | _ |  |  |
| Faurholt-Jepsen | _ |  | 0.75(0.49-1.15) |  |  |  |  |
| DDS | 0.96(0.87-1.05) |  | 0.63(0.44-0.90) |  | 0.55(0.32-0.94) |  |  |

TG=Triglycerides; LDL=Low density lipoprotein cholesterol; HDL=High density lipoprotein cholesterol; TC=Total Cholesterol; BP=Blood pressure; HbA1c=Glycated haemoglobin; ART=Antiretroviral therapy; CI =Confidence Interval; _ study did not have relevant data; GPC = General Population Cohort; DDS=Durban Diabetes Study; THUSA = Transition and Health during Urbanisation of South Africans; Africa centre = Africa Centre for Health and Population Studies . Note: the other studies bear the name of the collaborator(s) contributing data for pooled analyses.
